# Computational identification of tissue-specific transcription factor cooperation in ten cattle tissues

**DOI:** 10.1371/journal.pone.0216475

**Published:** 2019-05-16

**Authors:** Lukas Steuernagel, Cornelia Meckbach, Felix Heinrich, Sebastian Zeidler, Armin O. Schmitt, Mehmet Gültas

**Affiliations:** 1 Breeding Informatics Group, Department of Animal Sciences, Georg-August University, Margarethe von Wrangell-Weg 7, 37075 Göttingen, Germany; 2 Institute of Medical Bioinformatics, Goldschmidtstraße 1, University Medical Center Göttingen, Georg-August-University, 37077 Göttingen, Germany; 3 Center for Integrated Breeding Research (CiBreed), Albrecht-Thaer-Weg 3, Georg-August University, 37075, Göttingen, Germany; University of Florida, UNITED STATES

## Abstract

Transcription factors (TFs) are a special class of DNA-binding proteins that orchestrate gene transcription by recruiting other TFs, co-activators or co-repressors. Their combinatorial interplay in higher organisms maintains homeostasis and governs cell identity by finely controlling and regulating tissue-specific gene expression. Despite the rich literature on the importance of cooperative TFs for deciphering the mechanisms of individual regulatory programs that control tissue specificity in several organisms such as human, mouse, or *Drosophila melanogaster*, to date, there is still need for a comprehensive study to detect specific TF cooperations in regulatory processes of cattle tissues. To address the needs of knowledge about specific combinatorial gene regulation in cattle tissues, we made use of three publicly available RNA-seq datasets and obtained tissue-specific gene (TSG) sets for ten tissues (heart, lung, liver, kidney, duodenum, muscle tissue, adipose tissue, colon, spleen and testis). By analyzing these TSG-sets, tissue-specific TF cooperations of each tissue have been identified. The results reveal that similar to the combinatorial regulatory events of model organisms, TFs change their partners depending on their biological functions in different tissues. Particularly with regard to preferential partner choice of the transcription factors *STAT*3 and *NR*2*C*2, this phenomenon has been highlighted with their five different specific cooperation partners in multiple tissues. The information about cooperative TFs could be promising: i) to understand the molecular mechanisms of regulating processes; and ii) to extend the existing knowledge on the importance of single TFs in cattle tissues.

## Introduction

Regulation mechanisms of gene expression are of fundamental importance for different cellular processes, for instance, tissue development, differentiation or adaption to changing environmental conditions [1–3]. Today, it is well known that the precise and effective regulation of the transcriptional machinery in higher organisms is often achieved by the cooperation of transcription factors (TFs) [1, 2, 4]. Such cooperative TFs frequently bind to the regulatory regions of the DNA (promoters as well as enhancers) in a cell-type specific manner to govern a large spectrum of biological processes, e.g. cell-cycle or homeostasis [1, 3, 5]. The identification of such cooperative TFs in higher organisms is important to distinguish common biological processes from individual regulatory programs that control tissue specificity [5] (for more details see the reviews [3, 6]).

In the last years, several groups have successfully studied tissue-specific combinatorial gene regulation based on the complex interplay between multiple TFs in different organisms [5, 7–15]. A small representative number of these studies is presented in Table 1. In addition, a variety of databases like TransCompel [16], BioGRID [17], STRING [18], or TRRUST [19] have been created to store both experimentally verified and computationally predicted cooperativity of TFs, as well as proteins in general.

**Table 1:** Representative studies for the tissue-specific combinatorial gene regulation based on TF cooperations.

Authors	Synopsis of study	Type of data
Ament et al. [7]	Modeling of transcriptional network controlling mouse and human striatum as well as exploring the role of 48 TF-TF interactions in mouse models of Huntington’s disease	RNA-seq and microarray gene expression data
Sonawane et al. [5]	Investigation of cooperative TFs in regulatory networks for 38 human tissues	RNA-seq data from the Genotype-Tissue Expression project
Zeidler et al. [8]	Exploration of interacting TFs to understand the gene regulatory mechanisms during heart development	RNA-seq time series dataset including five time points
Song et al. [9]	Understanding and explanation of the role of 21 environmental stress related TF and their cooperativeness in the comprehensive regulatory network of *Arabidopsis thaliana*	Chip-seq and RNA-seq data
Rhee et al. [10]	Genome-wide analysis performed for *Drosophila melanogaster* in order to determine crucial biological functions of TF cooperations in tissue specification	RNA-seq data of 29 tissues and developmental time points from the modENCODE project
Nandi et al. [11]	Modeling of non-random functional dimers between the transcription factor MyoD and some muscle specific factors in the promoters of human genes	Human promoter sequences from the DBTSSs [20]
Laresgoit et al. [12]	Explanation of the essential role of the cooperation between transcription factors E2F2 and CREB for the regulation of transcriptional activity of cell cycle genes in mice	Data from ChIP-chip experiments
Myšičková et al. [13]	Systematic large-scale analysis for the characterization of tissue-specific TF interactions of 22 human tissues	Expressed Sequence Tags (EST) data
Girgis et al. [21]	Prediction of cis-regulatory motifs in 72 human tissues and identification of related TFs	Expression data from GNF Atlas
Hu et al. [15]	Systematic large-scale analysis for the identification of tissue-specific TF interactions for 79 human tissues	Gene expression data from GNF Atlas2 gene expression database (gnfAtlas2) [22]
Yu et al. [14]	Systematic large-scale analysis for the characterization of tissue-specific combinatorial gene regulation based on TF interactions for 30 human tissues	Tissue-specific genes from NCBI EST database

Despite the rich literature on tissue-specific cooperations of TFs in different organisms, as mentioned in Table 1, their importance in the gene regulatory mechanisms of underlying biological processes in the cattle genome has not yet been extensively studied. Until now, only few research groups have investigated the crucial role of (single) TFs in the cattle genome. For this purpose, Lim et al. [23] analyzed the promoters of differentially expressed genes of the Korean cattle breed Hanwoo and determined significant tissue-specific TFs for fat-, muscle-, and liver-tissues. Moreover, Bickhart et al. [24] performed a large-scale genome-wide analysis to predict 379333 transcription factor binding sites (TFBSs) and their associations with known SNPs by considering the promoters of 7764 annotated genes in the cattle genome. Recently, Weber et al. [25] examined feed conversion in Angus by analyzing RNA-seq data of metabolism related tissues (pituitary, visceral adipose, duodenum, liver and skeletal muscle). Applying partial correlation and information theory (PCIT) based methods, they constructed coexpression networks and determined the hub TFs, which act as important regulators in a tissue specific manner [25].

In order to address the limited knowledge available about crucial biological functions of tissue-specific TF cooperations in cattle, we analyzed in this study the promoter regions of tissue-specific genes (TSGs) of ten cattle tissues for the identification of their specific combinatorial gene regulation mechanisms. For this aim, using three publicly available RNA-seq datasets, we first identified a set of TSGs for each tissue according to their expression values and the significant TF cooperations for each tissue were determined using the PC-TraFF approach [1]. Subsequently, by applying the extension of the PC-TraFF approach (PC-TraFF^+^ [26]), the significant pairs have been assigned to two distinct groups as: i) TSG-set-specific TF cooperations; and ii) common (generally important) TF cooperations. As a result of our analysis, we obtained for each tissue a list of TSG-set-specific cooperative TF pairs which are likely to be fundamentally implicated in the regulation of transcriptional activity of a particular tissue. In the Result section of our study, we focused on these pairs and exemplarily explained their importance as well as potential roles in ten cattle tissues by providing further insight into the regulatory programs controlling specific biological processes such as tissue specificity or development.

## Materials and methods

### RNA-seq datasets

Datasets from three publicly available studies (described below) were selected for the identification of tissue-specific genes.

The first dataset has been published by Weber et al. [25] and consists of 16 samples from five cattle tissues (skeletal muscle, liver, visceral adipose tissue, pituitary and duodenum). The dataset contains of 17016 out of in total 24737 annotated cattle genes that have an expression value ≥ 0.2 (unit of expression: reads per kilobase of gene per million mapped reads, RPKM). In their study, Weber et al. described a gene *g*_*i*_ to be specific for the tissue *t*_*j*_, (*j* = 1, ⋯, 5) if the expression value of *g*_*i*_ in *t*_*j*_ is ≥ two thirds of *g*_*i*_’s accumulated expression values over all tissues. In total they identified 1026 genes as TSG: 285 genes for pituitary, 220 for skeletal muscle, 275 for liver, 33 for visceral adipose tissue, and 213 for duodenum.

In order to extend the number of tissues and increase the confidence in the TSGs, we included two further RNA-seq data sets available through the EBI Expression Atlas in our analysis [27]. The data from Merkin et al. (Accession: E-MTAB-2798) consists of 27 samples from nine tissues (brain, colon, heart, kidney, liver, lung, skeletal muscle, spleen and testis) and was part of a study on tissue-specific transcriptome variation across mammals [28]. The third dataset was created by Liao et al. (Accession: E-MTAB-2596) to examine duplicate genes in the cattle genome and their expression divergence. This dataset includes nine samples from seven cattle tissues (adipose, duodenum, hypothalamus, kidney, liver, lung, muscle) of beef cattle from Canada [29]. Merkin’s and Liao’s datasets contain expression values for 21414 and 20688 cattle genes, respectively, which are given as transcripts per million (TPM) values.

### Data processing

To deal with bias of genes with small TPM values during the TSG selection process, data processing analysis was conducted individually for each dataset. For this purpose, we first analyzed the distribution of TPM-values using density plots and established an expression threshold value of *τ* = 1.46, which indicates for datasets the local minimum of the density (see S1 Fig for the density plot of the TPM-values). Consequently, the genes with TPM values ≤ *τ* in all tissues are removed from Merkin’s and Liao’s RNA-seq datasets. The same threshold *τ* is also used for the selection of expressed TFs in Results section.

### Tissue-specific gene selection

Following the TSG description strategy of Weber et al., [25] we identified the TSGs in Merkin’s and Liao’s RNA-seq datasets. As a result, we obtained the TSG-sets for 13 unique cattle tissues based on all three RNA-seq datasets. However, due to the inconsistency between the RNA-seq datasets, we eliminated all TSGs for the brain tissues from our analysis, since each experiment examined different parts of the brain (pituitary in Weber’s dataset, hypothalamus in Liao’s dataset and unspecified brain tissue in Merkin’s dataset). Finally, following the study of Gusev et al. [30], we determined the common TSGs of a tissue, if it was included in more than one experiment/dataset, to compensate the effects of different experimental conditions and the variation between individual animals under study.

The selection of TSG-sets for each tissue was performed as follows:

1. The TSG-set for liver contains all liver specific genes common in all three datasets.
2. The TSG-sets for lung and kidney contain their corresponding specific genes common between Merkin’s and Liao’s datasets.
3. The TSG-sets for duodenum, muscle-, and adipose-tissue are from Weber’s dataset.
4. The TSG-sets for heart, colon, spleen and testis are from Merkin’s dataset.

### Identification of cooperative TFs

We applied the PC-TraFF [1] and PC-TraFF^+^ [26] methodologies to the above defined TSG-sets for the identification of tissue-specific TF cooperations. The theory behind the approaches used in this sections are detailed in [1] and [26].

PC-TraFF is an information theory method that applies pointwise mutual information (PMI) for the detection of cooperative TFs based on the co-occurrence of their binding sites in the regulatory regions of genes. Its algorithm consists of six steps and requires regulatory sequences, a list of position weight matrices (PWMs) and pre-defined distance preferences of TFBSs as input parameters:

- **Regulatory sequences**: using the UCSC genome browser [31], we extracted for each gene in the TSG-sets its corresponding promoter sequence covering the −500 to 100 bp regions relative to transcription start sites.
- **PWMs and TFBS detection**: we used non-redundant vertebrate PWMs from the TRANSFAC database (release 2018.1) [32] and employed the Match [33] program with these PWMs by setting its profile parameter “minSum: cut-offs for minimizing the sum of false positive and false negative rate” for prediction of potential TFBSs in promoter sequences. The selection process of PWMs for each tissue is explained in the Results section.
- **Pre-defined distances**: as a prerequisite, the PC-TraFF algorithm needs user-specified minimum and maximum distances which are necessary for the construction of TFBS pairs. As a result, two TFBSs are considered to be able to form a dimer if their distance preferences satisfy the user-specified distances. In this study, we use the recommended distance values and set the minimum distance ≥ 5 and maximum distance ≤ 20.

#### Significant TF cooperations

The PC-TraFF algorithm provides for each TF-pair *t*_*a*_ and *t*_*b*_ a PMI(*t*_*a*_; *t*_*b*_)-value based on the co-occurence frequencies of their TFBSs in the sequences of interest. In a final step, the algorithm transforms the PMI(*t*_*a*_; *t*_*b*_)-values into *z-scores* and the cooperation between *t*_*a*_ and *t*_*b*_ is considered to be *statistically significant* if they have a *z-score* ≥ 3.

#### TSG-set-specific TF cooperations

For the division of significant TF cooperations into the two categories of TSG-set-specific and common (generally important) cooperations, we further applied the PC-TraFF^+^ algorithm, which is an extension of the PC-TraFF approach. The main methodology of PC-TraFF^+^ is a heuristic approach that estimates the level of background cooperation (AVG(PMI(*t*_*a*_; *t*_*b*_))-value) of any TF pair based on simulated sequence sets. Afterwards, the AVG(PMI)-values are subtracted from the initial significant PMI-values:

$${\text{PMI}}^{\text{specific}}\left(t_{a};t_{b}\right)=\text{PMI}\left(t_{a};t_{b}\right)-[\left(1+\alpha \right)\times \text{AVG}(\text{PMI}(t_{a};t_{b}))],$$ (1)

where *α* ∈ [−1, + 1] is a preassigned scaling factor which is used to control the influence of the background level. If *α* = −1, there will be no differentiation between the TSG-set-specific and common (generally important) TF cooperations. On the other hand, setting *α* ≥ 0 results in the enlargement of subtracted background level which will lead to a more strict separation process. Consequently, a positive PMI^specific^-value indicates the TSG-set-specific cooperation of a TF pair, whereas a PMI^specific^-value ≤0 refers to a common cooperation of corresponding TFs that could play a generally important role in regulatory programs.

**Fig 1:**
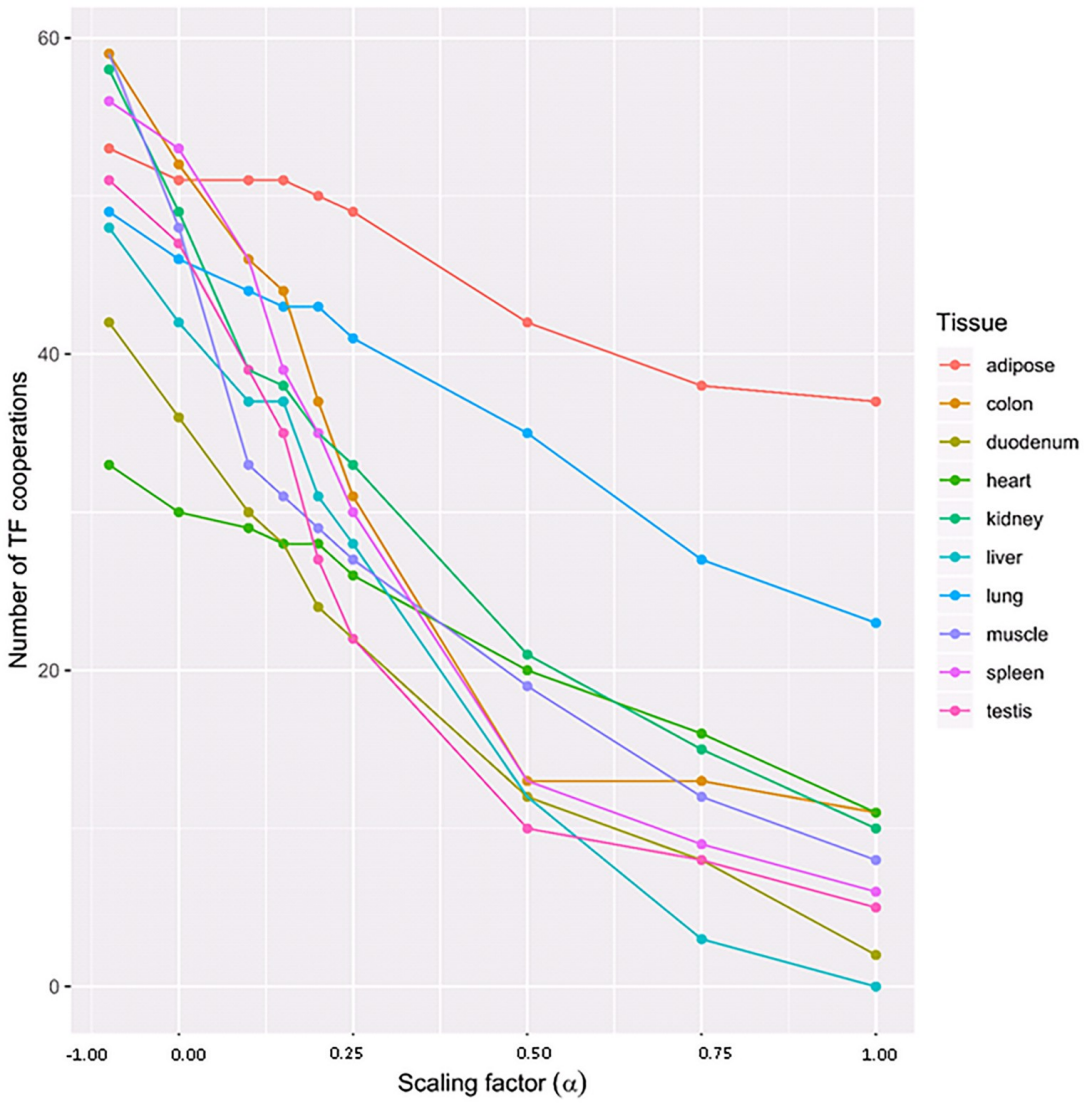
Number of tissue-specific TF cooperations identified by the PC-TraFF^+^ algorithm with different *α*-values. The subtracted background grows with *α*, thus reducing the number of specific cooperations.

In order to determine the most convenient/suitable value of *α* in our analysis, we followed the recommendation of the PC-TraFF^+^ approach and systematically tested different *α*-values for the assessment of their influence on the ratio between tissue-specific and common (generally important) TF cooperations. As shown in Fig 1, the impact of *α* itself is not linear and highly tissue (TSG-set) dependent. Going through different *α*-values, we established that setting *α* > 0.5 results in a dramatic decrease in the number of identified specific pairs of multiple tissues (see Fig 1). For this reason, we set *α* = 0.5 for our analysis.

It is important to note that the key concept of the PC-TraFF^+^ approach provides for those significant TF pairs, which are very sensitive to the nucleotide composition and the position of their binding sites in the sequences of interest, remarkably small AVG(PMI)-values and thus positive PMI^specific^-values. As a result, a significant TF pair is defined to be specific if its corresponding PMI^specific^-value is > 0 for the dataset under study.

## Results

**Fig 2:**
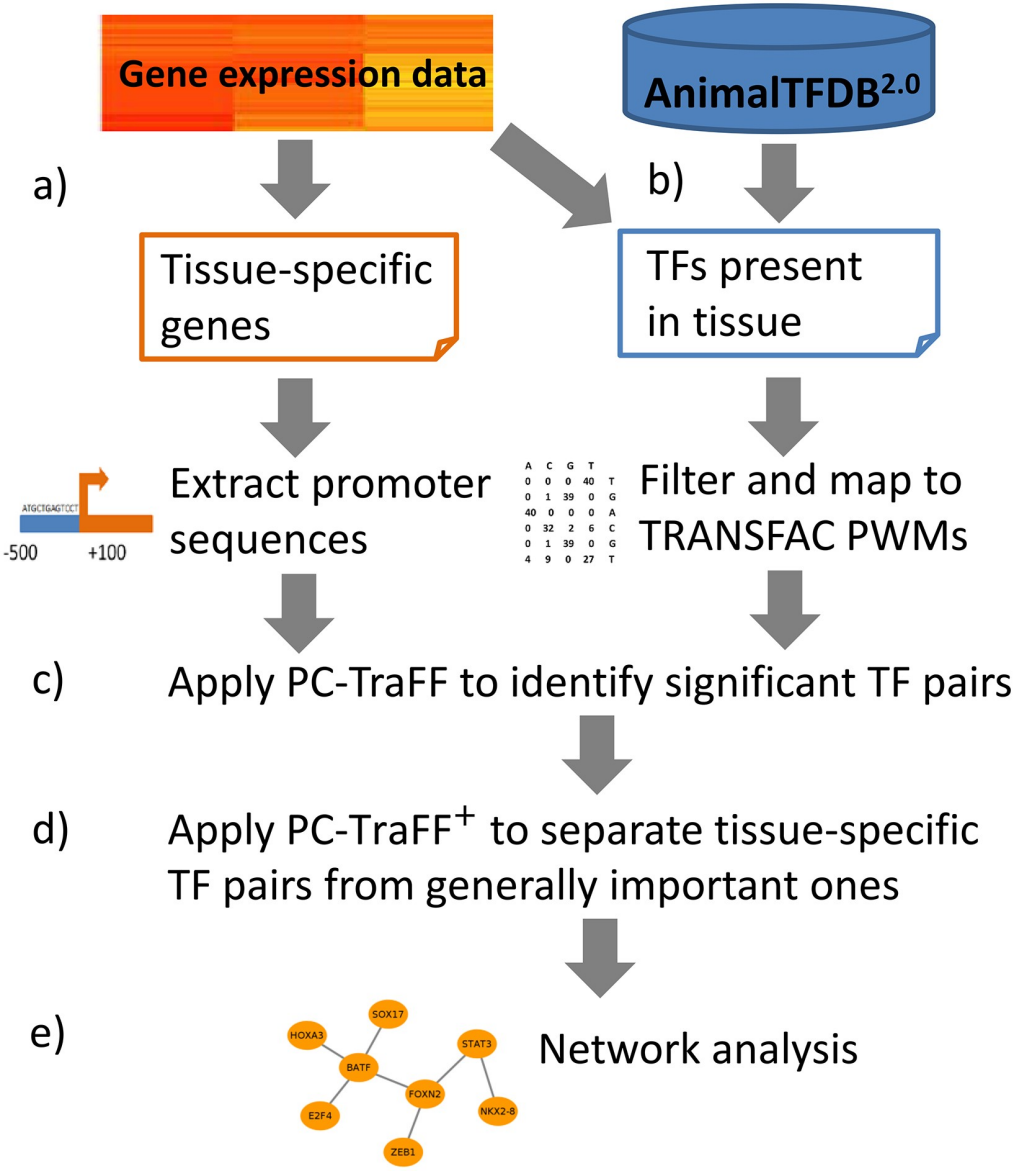
Flowchart of analysis procedures. (a) Identification of tissue-specific genes from RNA-seq data and extraction of promoter region of genes. (b) Identification of TFs expressed for each tissue in RNA-seq data. (c) Application of PC-TraFF [1]. (d) Application of PC-TraFF^+^ [26]. (e) Reconstruction of tissue-specific TF-TF cooperation networks.

In this study, to identify tissue-specific transcription factor cooperations, we analyzed tissue-specific genes (TSGs) of ten cattle tissues by employing PC-TraFF and its extension (PC-TraFF^+^) [1, 26]. For this purpose, we first collected publicly available RNA-seq datasets and we defined sets of tissue-specific genes following the TSG description of Weber et al. An overview of the TSGs is given in Table 2 (for a list of TSGs see S1 Table). In the next step the PC-TraFF algorithm was applied to the promoter sequences of the TSG-sets to identify significant TF cooperations. However, due to common regulatory programs between tissues as well as the different properties of promoter sequences like their GC content, dinucleotide occurrence or mononucleotide composition—referring the order and frequencies of individual nucleotides in sequences [26] -, some TF pairs have been ubiquitously determined as significant for multiple tissues. In order to eliminate negligible pairs (ubiquitously appearing, generally important ones) and to emphasize the roles of tissue-specific TF pairs, we applied the extension approach PC-TraFF^+^ [26]. Consequently, we determined tissue specific TF-cooperations and focused on these TF pairs in our further analysis to understand the molecular mechanisms of tissue-specific regulatory processes. The overall analysis procedures are outlined in Fig 2.

**Table 2:** Numbers of TFs and tissue specific genes under study.

Tissues	Number of TSGs	Number of TFs
Heart	58	397
Lung	104	394
Liver	153	312
Kidney	163	395
Duodenum	213	285
Muscle tissue	220	407
Adipose tissue	33	383
Colon	213	369
Spleen	215	343
Testis	1958	297

### Selection of expressed TFs in tissues

As a prerequisite of the PC-TraFF approach, a library of position weight matrices (PWMs) is required to predict the putative binding sites of TFs in the promoter sequences. To fulfill this criterion, we first determined for the cattle TFs from AnimalTFDB 2.0 [34] the expression values (transcript per million (TPM) values) of the respective TF genes in the RNA-seq data of each tissue. Subsequently, using the threshold *τ* = 1.46 established in Material and Methods section, we discarded all TFs with an expression value of smaller than *τ*. The remaining TFs were mapped to PWMs stored in the TRANSFAC database [32]. In order to avoid redundancies, Pearson correlations between these PWMs were computed, the PWMs were clustered with hierarchical clustering based on their correlation coefficients and the PWM with the highest information content in each cluster was chosen as representative. An overview of the number of TFs of interest is given in Table 2.

**Fig 3:**
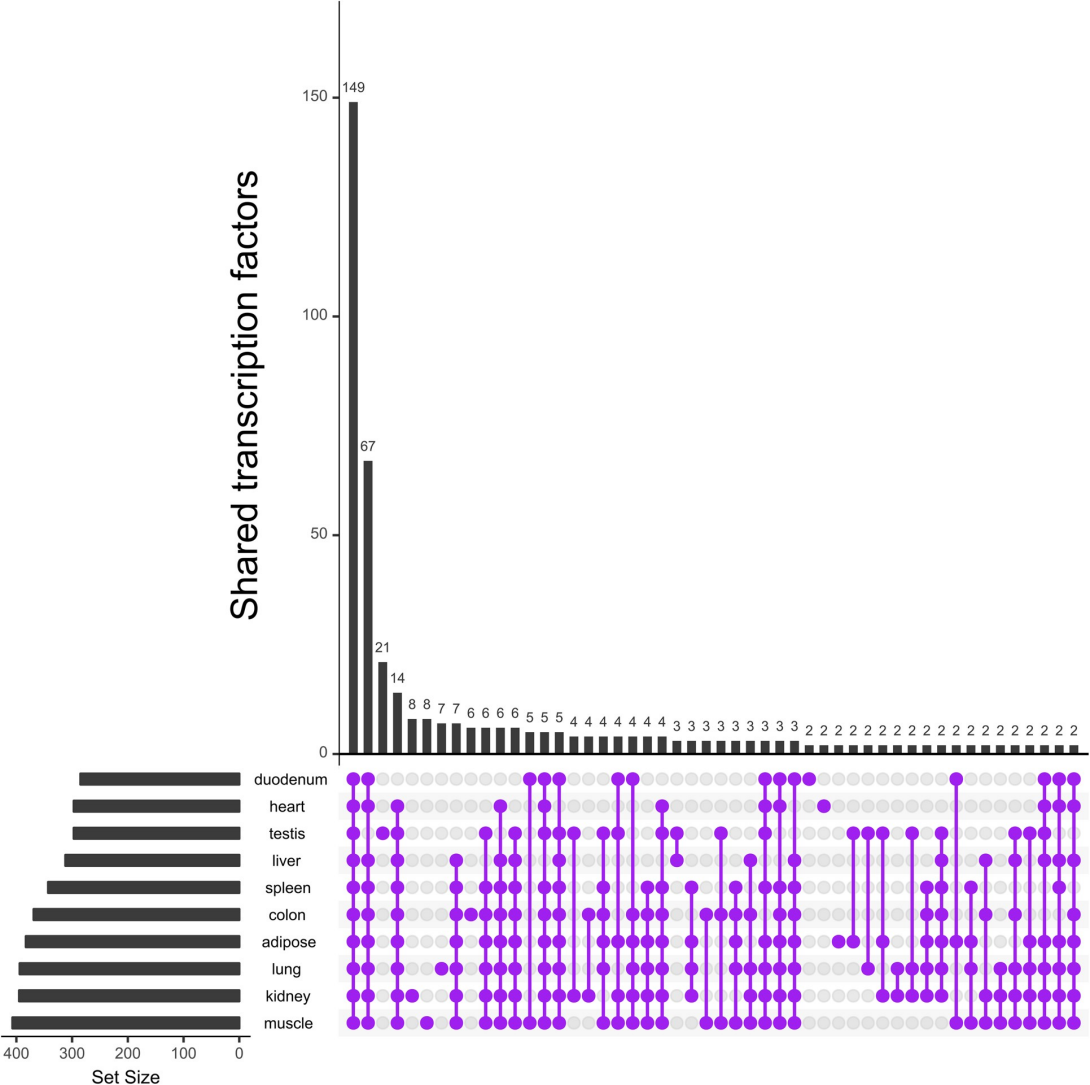
Occurrence of TFs present in the tissues. Number of TFs with an expression value ≥ *τ* and their overlap between ten tissues represented in matrix layouts using the UpSet technique [35]. Purple circles in the matrix layout are related to the tissues that are part of the intersection. For the sake of clarity not all intersections are displayed.

To gain more insight into the TFs of interest, we evaluated their overlap between tissues. Fig 3 shows that the majority of TFs are present in all ten tissues, and only a few are unique to each tissue.

### Identification of tissue specific TF-cooperations

Applying the PC-TraFF algorithm to promoter sequences of TSGs, we identified for each tissue significant cooperative TF pairs. However, due to the common regulatory processes carried out by several tissues, the significant TF pairs are partially overlapping. In order to separate TSG-set-specific TF cooperations from the common ones, we additionally employed the PC-TraFF^+^ [26] approach and eliminated 322 negligible pairs from our analysis. The numbers of significant and TSG-set-specific TF pairs are given in Table 3 (the whole list of pairs for each tissue can be found in S2 Table).

**Table 3:** Numbers of cooperative TF pairs identified for each tissue as significant by PC-TraFF and TSG-set specific by PC-TraFF^+^.

	Number of cooperative TF pairs	
Tissue	Significant pairs	TSG-set-specific pairs
Heart	36	22
Lung	49	35
Liver	48	12
Kidney	62	25
Duodenum	50	13
Muscle tissue	63	21
Adipose tissue	59	48
Colon	68	15
Spleen	56	13
Testis	44	9

**Fig 4:**
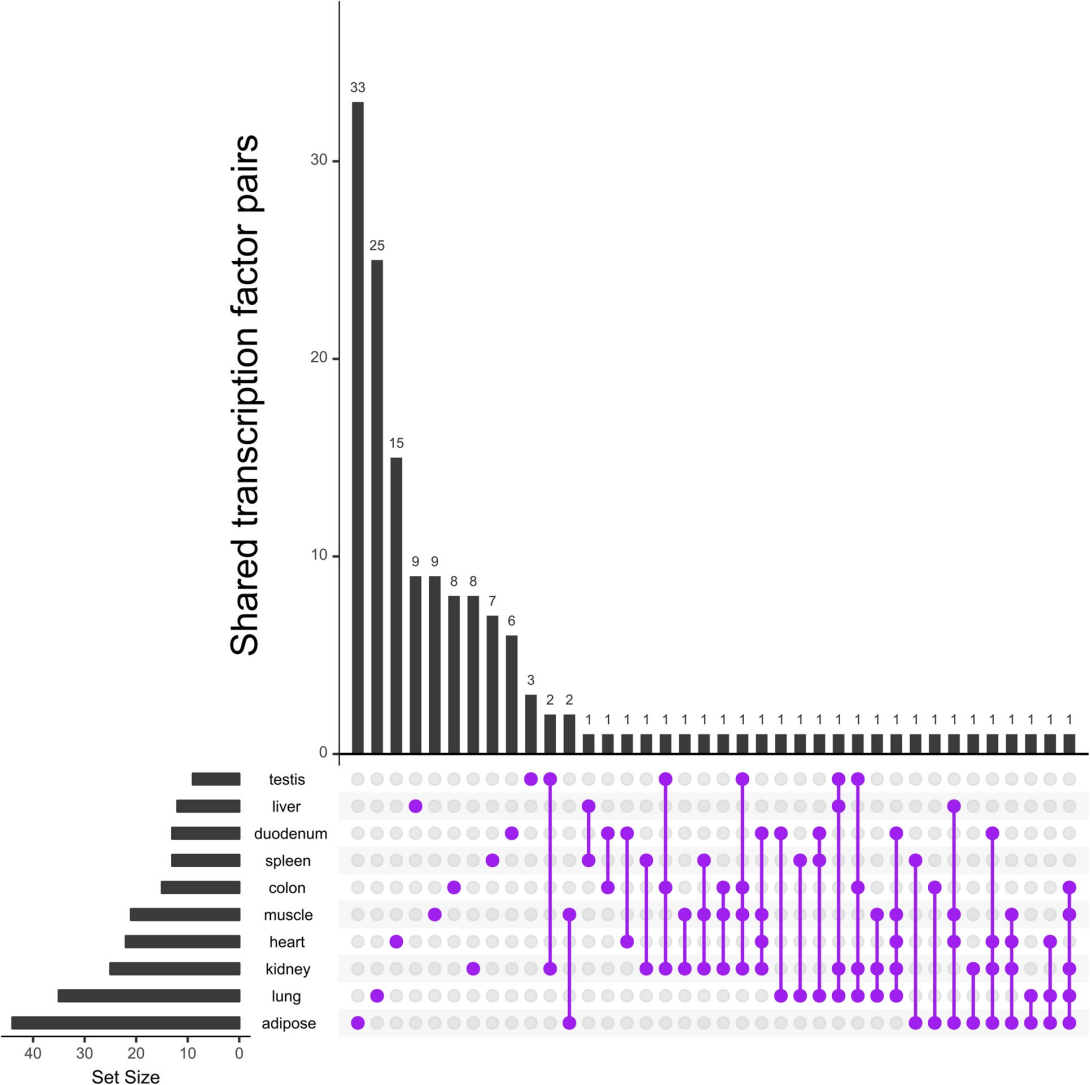
Occurrence of TSG-set-specific TF cooperations identified by PC-TraFF^+^ approach in ten tissues. Number of TF cooperations and their overlap between tissues represented in matrix layouts using the UpSet technique [35]. Lines with purple circles in the matrix layout show the tissues with overlapping TF cooperations. For the sake of clarity not all intersections are displayed.

The analysis of TSG-set-specific TF pairs reveals that although the overlap of single TFs in multiple tissues is high (see Fig 3), the intersection of specific TF pairs is remarkably small (see Fig 4). In total, 213 out of 535 cooperative pairs have been assigned as tissue specific by PC-TraFF^+^. Further, 123 of these specific TF pairs have been determined as TSG-set-specific for a certain tissue and the remaining pairs have been determined as specific for at least two tissues. Interestingly, as shown in Fig 4, among all tissues only two combinations share more than one TF pair as TSG-set-specific. In particular, the transcription factor pairs *NR*2*C*2—*SP*1 and *SP*2—*TEAD*2 were found to be specific in colon, muscle, and kidney, while the TF pairs *BCL*6*B*—*SMAD*5 and *GTF*2*I*—*STAT*3 have been identified as specific for muscle and adipose, respectively. Among these transcription factors, *BCL*6*B* is a transcriptional repressor, which plays a role in immune responses of T-cells [36]. Its cooperation partner *SMAD*5 is a family member of the *SMAD* factors [37] that plays a role in the transforming growth factor-*β* (*TGF*-*β*) signaling pathway, however they are also known to affect various other regulation mechanisms such as pathways controlling cell-cell adhesion [38]. Further, *SMAD*5 is an important regulator in pathways controlling muscle mass, as it transmits the signal of the growth factor Bone morphogenetic protein (*BMP*) [39]. Additionally, *SMAD* factors are reported to affect adipocyte differentiation and metabolism [40].

The factor signal transducer and activator of transcription 3 (*STAT*3) is a family member of the *STAT* factors [37] and is involved in immune signaling, e.g. cytokine signaling [41]. In cattle *STAT*3 is known to mediate signals during inflammatory response and to be relevant for fertility and embryo survival rate [42, 43]. It is activated by phosphorylation through Janus-kinases and potentially participates in adipogenesis and body weight homeostasis [44, 45]. Its cooperation partner *GTF*2*I* was at first identified to bind to the initiator element (Inr) of a multitude of promoters. Nowadays, the factor is known to be important for transcriptional initiation of *TATA*-less promoters and besides this general task, it is involved in cell-type specific regulatory processes of gene expression. The interplay between the *GTF*2*I* and *STAT* factors has been identified for the regulation of the *c*-*fos* promoter where the binding of *STAT* factors (*STAT*1 or *STAT*3) is required to achieve the maximal activity of the promoter bound *GTF*2*I* factor. Further, *STAT*3 and *GTF*2*I* have been shown to dimerize in vivo indicating that both factors bind next to each other on DNA and thereby physically interact with each other in order to activate *c*-*fos* gene expression [46, 47].

The top three TSG-set-specific TF pairs for each tissue are presented in Table 4. Several TFs participate in different pairs across the different tissues and form specific cooperative dimers according to their biological functions. In particular, the transcription factors *NR*2*C*2 and *STAT*3 form cooperative pairs with different partners across the five different tissues. The factor *NR*2*C*2 is a member of the *RXR*-related receptors family [37] and can act as a repressor or activator of various signaling pathways, specifically those of other TFs of the nuclear receptors with *C*4 zinc fingers class [48]. *NR*2*C*2 preferentially binds to target sites in open chromatin regions and is known to affect cell type specific genes as well as RNA metabolism and protein translation [49].

**Table 4:** Top three TSG-set-specific cooperative TF pairs of each tissue.

Tissue	Top three specific TF pairs
Heart	[*BCL*6*B*—*NR*2*C*2]; [*NKX*2 − 5—*HMGA*1]; [*FOXN*2—*TCF*12]
Lung	[*SP*3—*NR*2*C*2]; [*SMAD*5—*TEAD*2]; [*BCL*6*B*—*NR*2*C*2]
Liver	[*SMARCC*2—*STAT*3]; [*ZBTB*7*A*—*SP*2]; [*HOXB*3—*E*2*F*4]
Kidney	[*KLF*4—*NR*2*C*2]; [*SP*1—*NR*2*C*2]; [*SP*3—*NR*2*C*2]
Duodenum	[*SMAD*1—*HMGA*1]; [*KLF*4—*NR*2*C*2]; [*MYF*6—*NR*2*C*2]
Muscle	[*TCF*3—*KLF*12]; [*SP*2—*TEAD*2]; [*KLF*4—*NR*2*C*2]
Adipose	[*TCF*12—*STAT*3]; [*GTF*2*I*—*STAT*3]; [*KLF*12—*TGIF*2]
Colon	[*HAND*1—*STAT*3]; [*TCF*3—*STAT*3]; [*SMAD*5—*CTCF*]
Spleen	[*ZBTB*7*A*—*TEAD*2]; [*NKX*2 − 5—*IRX*6]; [*HMGA*1—*BATF*]
Testis	[*KLF*4—*SP*1]; [*KLF*4—*KLF*4]; [*KLF*4—*TEAD*2]

The pairs are sorted in ascending order based on the their *z-scores* provided by PC-TraFF^+^.

In addition, other crucial TFs with varying partners have been identified: *SP*1, *SP*2 and *SP*3 are family members of the three-zinc finger Krüppel-related factors [37] and cooperate with *NR*2*C*2, *ZBTB*7*A*, and *TEAD*2 in several tissues (see Table 4). The factor *SP*1 is a well-known TF, which activates genes with functions in housekeeping, tissue-specificity, and cell cycle-regulation [50]. *SP*2 is reported to bind to T-cell promoters, which could indicate an immune response related function [51]. *SP*3 resembles *SP*1 closely in its structure, but can affect the transcriptional regulation of genes in different ways [52]. For example during the *PKR* gene activation, *SP*3 can modulate expression mediated by interferons, while *SP*1 is responsible for the basal transcription [53]. Both *SP*1 and *NR*2*C*2 are reported to bind RB binding protein 4 (*RBB*4), a chromatin remodeling factor, which might mediate this cooperation [54, 55].

Further analysis of individual TF pairs in Table 4 demonstrates that there is literature support for the interaction between some cooperative pairs although our prediction of TF cooperations does not relate to their direct physical interactions. For example, the cooperative TFs *SMAD*5 and *CTCF* in colon are reported to bind to the same promoter regions to interact in transcriptional regulation in mammals and *Drosophila melanogaster*, where *CTCF* is important for the recruitment of *SMAD* factors [56, 57]. Another remarkable pair found in cattle heart tissue is *HMGA*1—*NKX*2-5. The homeodomain factor *NKX*2-5 is a well-studied TF which plays essential roles in healthy heart development and disease [58, 59]. Being a member of high-mobility group A (*HMGA*) non-histone chromosomal proteins, *HMGA*1 is an important TF during heart development and growth as well as cardiomyocytic cell growth regulation [8, 60, 61].

### Analysis of tissue-specific TF cooperation networks

**Fig 5:**
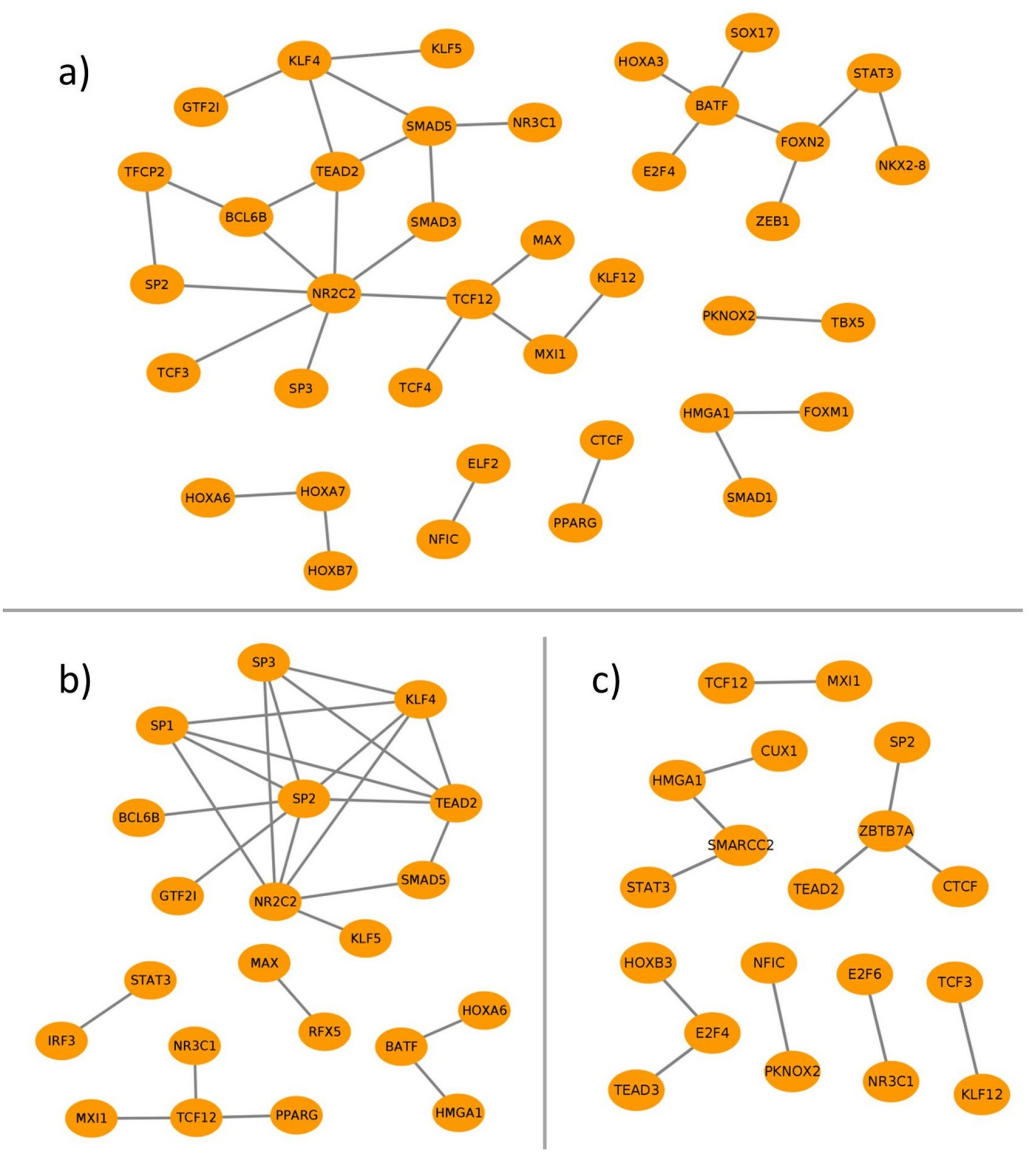
Cooperation networks for the TSG-set-specific TF pairs of (a) lung-, (b) kidney- and (c) liver-tissue.

To further establish the potential role of TSG-set-specific TF pairs, we constructed, similar to our previous studies [1, 8, 26], a cooperation network for each tissue, in which the nodes depict the TFs and edges refer to their cooperativity (see Fig 5). On the one hand, these networks are essential to monitor the preferential partner choice of TFs in different tissues. On the other hand, they highlight the functional dimers or high order complexes of TFs as well as the hub TFs which could provide crucial information for explaining the underlying mechanisms of regulatory events.

It is important to note that in the following analysis we have mainly focused on the TF cooperation networks of three cattle tissues, namely lung, kidney and liver, because of the higher confidence in their TSGs (see Materials and methods). The cooperation networks of the remaining seven tissues can be found in S2 Fig.

Fig 5 presents the cooperation networks of lung-, kidney-, and liver tissues based on their specific-TF pairs, which consist of 35, 25 and 12 cooperative pairs for each tissue, respectively. In comparison to liver and kidney, the network of lung tissue consists of more pairs, although the number of lung specific genes is smaller than those of kidney and liver. This result is in line with our previous studies [1, 8, 26] and indicates that the number of TSG-set-specific TF pairs depends neither on the number of TSGs nor on the number of TFs under study (for the numbers see Table 2).

A general analysis of the networks indicates that the transcription factor *NR*2*C*2 forms a hub in the networks of lung- and kidney specific-TF pairs, with a degree of seven and four respectively (Fig 5a and 5b). Among its partners, only its cooperations with *SP*2 and *SP*3 have been simultaneously identified as specific for both tissues. Additionally, an interesting cooperation is monitored between *NR*2*C*2 and *SMAD* factor family members: while *NR*2*C*2 cooperates with *SMAD*3 in lung tissue, the pair *NR*2*C*2-*SMAD*5 is determined as tissue-specific in kidney.

Other remarkable cooperations in the network of lung tissue are observed between the following TFs: *PPARG*—*CTCF*, *KLF*4—*SMAD*5, *KLF*4—*TEAD*2, and *SMAD*5—*TEAD*2. The factor peroxisome proliferator-activated receptor gamma (*PPARG*) is reported to be an important regulator in adipogenesis. The cooperation of *CTCF* with *PPARG* and its effects on the transcriptional activity of *PPARG* are well documented in [62, 63]. *SMAD* factors are known to form complexes with *KLF*4 and to mediate its phosphorylation, which is necessary for the recruitment of other factors such as *PPARG* [64, 65]. In human stem cells transcriptional complexes, involving *TEADs* and *SMAD* factors that link the *TGF*-*β* and the hippo signaling pathway were described [66]. Additionally, both *KLF*4 and *TEADs* are reported to be partners of yes-associated proteins (*YAPs*), which are important regulators in the hippo signaling pathway [66, 67]. This supports the hypothesis of a multi-factor complex that has a tissue specific role in the lung, potentially affecting cell proliferation or differentiation as the hippo and *TGF*-*β* regulatory pathways suggest.

A closer look at the cooperation network of kidney-specific TF pairs (Fig 5b) reveals that the largest hubs *NR*2*C*2 and *SP*2 form a cooperative dimer which also has been identified as specific in four other tissues (heart, lung, duodenum, and muscle) (see S2 Fig). *NR*2*C*2 is a receptor of various hormones such as androgenes and it is known to bind and orchestrate transcriptional regulation with the Hepatocyte nuclear factor 4 alpha (*HNF*4*A*), which plays an essential role in kidney cell proliferation and development [68, 69]. *SP*2 could indicate an immune response related function [51]. *KLF*4 was detected to cooperate with *NR*2*C*2, *SP*1 and *SP*2 and it belongs to the same family of three-zinc finger Krüppel-related factors as *SP*1 and *SP*2 [37]. The cooperation of *SP*1 and *KLF*4 is experimentally confirmed by a known protein-protein interaction (PPI) of these TFs, which cooperate when binding to the transforming growth factor *TGF*-*β* control element in promoters [65]. Another interesting hub in this network (Fig 5b) is *TCF*12 that is cooperating with *PPARG*, MAX interactor 1 (*MXI*1) and the glucocorticoid receptor (*NR*3*C*1). *TCF*12 in human is involved in the repression of E-cadherin [70], which plays essential roles in healthy animal tissue morphogenesis and development [71–74]. The role of *TCF*12 as a hub in our network suggests participation in other regulation mechanisms as well, e.g. by its cooperation with *PPARG*, which is associated with insulin sensitivity and other metabolic pathways in kidney [75]. The cooperations of *TCF*12 with *MXI*1 as well as *MAX* were detected to be specific for cattle lung tissue as well. The factors *MAX* and *MYC* dimerize to act in the transcriptional regulation of cell proliferation and *MXI*1 inhibits this mechanism by competing with *MYC* for *MAX* binding sites and additionally repressing *MYC* transcription [76, 77]. Through its cooperation with *MXI*1 and *MAX*, *TCF*12 might influence this important transcriptional repression mechanism in some cattle tissues.

The liver-specific TF pairs constitute the smallest cooperation network in this study, which includes 12 cooperative pairs in multiple unconnected subgroups (see Fig 5c). Having three different cooperations with *CTCF*, *TEAD*2, and *SP*2, the transcription factor *ZBTB*7*A* (zinc finger and BTB domain-containing protein 7A) forms the largest hub in this network. *ZBTB*7*A* is reported to bind to many promoter sequences in the human genome and it cooperates with different TFs and proteins that influence DNA accessibility to modulate gene regulation [78]. Its cooperation partner *CTCF* interacts with the chromatin structure in various ways [79] and can bind to the co-repressor *SIN*3*A*, which plays a role in the recruitment of histone-deactylases [80]. Interestingly, *SIN*3*A* can also bind to *ZBTB*7*A* in human, which might suggest a potential function of it as a co-factor in this cooperation [81].

## Discussion

Today, it is widely known that the cooperation of TFs is crucial for the precise orchestration of tissue-specific genetic programs and/or transcriptional regulation in cells [1, 5]. Until now, several studies have shown that the TF partnership is accomplished through a non-random process which depends strongly on their specific roles in different biological processes as well as on the cellular context [1, 5, 6, 11, 14, 21, 26]. For instance, modeling gene regulatory networks of 38 human tissues, Sonawane et al. [5] recently pointed out the dimerization of TFs in the regulatory events of these tissues and recognized the specific partner alterations of TFs that contribute to tissue specificity by coordinating distinct regulatory processes. A comparable study has been performed by Rhee et al. [10] to address the crucial roles of TF cooperations in the transcriptional regulatory network of *Drosophila melanogaster* (for an overview see Table 1). To this end, Amoutzias et al. [6] discussed in their review that knowledge about cooperative TFs is helpful for understanding of different disease mechanisms as well as in drug development.

However, regarding cattle tissues only limited information about TF cooperativity is available. In this study, we addressed this need and performed a comprehensive study for ten cattle tissues which could aid researchers to create novel hypotheses for transcriptional regulation as well as provide comparability with model organisms. For this aim, we have analyzed three publicly available RNA-seq datasets and found that only three out of ten tissues have multiple TSG-sets with overlapping genes. To increase the quality and simultaneously eliminate the differences of their TSG-sets, we considered the intersection between the TSGs obtained for a certain tissue as suggested in [30].

For the identification of tissue-specific TF-cooperations, the PC-TraFF and PC-TraFF^+^ approaches have been applied to promoter sequences of TSGs. However, due to their underlying methodologies, the prediction performance of both algorithms is heavily influenced by the putative TFBSs predicted by using PWM libraries. Stormo et al. [82] as well as Whitfield et al. [4] have pointed out that computational TFBS predictions using PWMs is a very effective and established method, but it suffers from high rates of false positive predictions. To reduce this to some extent in our analysis, a fundamental step for the construction of tissue-specific PWM libraries is the inclusion of TF genes with expression values ≥ *τ* in a tissue (see Results section). While the analysis of expressed TF genes provides crucial information about their comparability and presence in multiple tissues, the usage of their associated PWM libraries leads to the reduction of false positive TFBS predictions in the identification of tissue-specific TF cooperations.

Our results suggest that the consideration of single TFs, whose majority is present in all tissues under study, appears to be insufficient for the differentiation of common and specific regulatory programs (see Fig 3). Several studies show that in higher organisms TFs have to form cellular context-dependent cooperations rather than acting alone during, for instance, tissue differentiation and development [1, 5, 6]. Similar to previous studies based on model organisms, our results further show that TFs switch their partners to specify their biological functions depending on the tissue type, for example the transcription factor *STAT*3 exhibits specific (different) cooperation partner preferences on the level of transcriptional regulation of lung, kidney and liver (see Fig 5a, 5b and 5c). The partner choice of *STAT*3 might explain its potentially specific functions in gene regulatory mechanisms controlling tissue specificity as well as development [41]. *Interferon regulatory factor 3* (*IRF*3) was found to cooperate with *STAT*3 in kidney. *IRF*3 is a master regulator activating interferons or interferon stimulated genes, which activate Janus-kinases that subsequently phosphorylate *STAT* factors [83, 84]. *STAT* factors can bind to the response elements of interferon stimulated genes [85], which could indicate that *IRF*3 and *STAT*3 cooperate to regulate genes activating *STAT*3. In cattle liver, *STAT*3 cooperates with *SMARCC*2, a member of the *SWI*/*SNF* chromatin remodeling complex that can bind to various mammalian promoters and could be important for the liver-specific role of *STAT*3 [86]. Furthermore, *STAT*3 collaborates in lung with *forkhead* *box* *N*2 (*FOXN*2) and *NK*2 *homeobox* 8 (*NKX*2-8), which is a known lung developmental transcription factor that exhibits gene expression patterns related to *STAT* factors and Janus kinases pathways [87]. In general, *STAT*3 demonstrates the advantage of the employed method: it is present in all tissues, but the identification of its cooperations is necessary to uncover its tissue specific roles. Another interesting TF in the cooperation networks is *NR*2*C*2 which occurs in eight different tissues with multiple overlapping partners (see Fig 5a and 5b, and S2 Fig for the remaining six tissues). Despite the overlap between its partners, the *NR*2*C*2-dependent regulation processes of these tissues are characterized by a unique set of its partner TFs. On the one hand, it forms dimers with the three-zinc finger Krüppel-related factors (*SP*1, *SP*2, *SP*3 and *KLF*4, *KLF*5) in seven different tissues and with *SMAD* factors in five tissues. On the other hand, *NR*2*C*2 forms exclusive dimers, for example with: i) *TCF*12 and *TCF*3 in lung; ii) *MAF* in adipose tissue; iii) *IRF*3 in muscle tissue; iv) *MYF*6 in duodenum; v) *MXI*1 in spleen.

Notwithstanding the limited availability of tissue specific RNA-seq datasets in cattle compared to other organisms such as human or mouse, taken together, our results provide a comprehensive overview of the specific regulatory processes in different cattle tissues. This knowledge is required to better understand the regulatory mechanisms of biological processes during development, cell cycle or different diseases. In addition, focusing on the specific partner choice of TFs (e.g., *NR*2*C*2 and *STAT*3) highlights the need for our analysis to identify cooperative TF pairs in order to amplify the findings on the importance of single TFs for cattle tissues reported in [23–25].

## Conclusion

In this study, we aim to contribute to the understanding of tissue-specific combinatorial gene regulation mechanisms by addressing the limited knowledge available about crucial biological functions of tissue-specific TF cooperations in cattle. Specific functions of different tissues and their expressional regulation are largely dependent on the complex interplay between TFs. By forming dimers or high order complexes, TFs have to act with their non-random cooperation partners in higher organisms to ensure coordinated cellular processes in response to environmental stimuli as well as tissue-specificity. In order to explore complex interplay between TFs, we performed a comprehensive analysis using PC-TraFF and PC-TraFF^+^ approaches and identified tissue-specific TF cooperations in ten cattle tissues that are essential for directing the specific transcriptional program of tissues. The results show that similar to the combinatorial regulatory events of model organisms, TFs switch their partners depending on their biological functions in activation or repression of tissue specific genes. Furthermore, we highlighted the preferential partner choice of TFs in different tissues using cooperation networks which could aid researchers to get a better understanding for the underlying mechanisms of regulatory events as well as to generate new hypotheses regarding the molecular mechanisms of regulating processes in cattle tissues. On the top of that, the knowledge about TF cooperation could complement previous studies which mainly focused on the effect of single TFs.

## Supporting information

S1 Fig Density of expression values. (PDF)

S2 Fig Tissue-specific TF cooperation networks of seven cattle tissues. (PDF)

S1 Table Lists of tissue-specific genes. (CSV)

S2 Table Lists of significant and specific TF cooperations per tissue. (CSV)
