# Supplementary material for: Computational identification of tissue-specific transcription factor cooperation in ten cattle tissues
Source: PLoS One. 2019 May 16;14(5):e0216475. doi: 10.1371/journal.pone.0216475 (PMC6522001; doi:10.1371/journal.pone.0216475)

Density of expression values as logarithmized TPM-values aggregated over all genes and tissues for the identification of a threshold to clarify TF genes and TSGs as expressed. The plot shows largely bimodal distributions with a common minimum at TPM=1.46. Consequently, the local minimum was chosen as threshold.

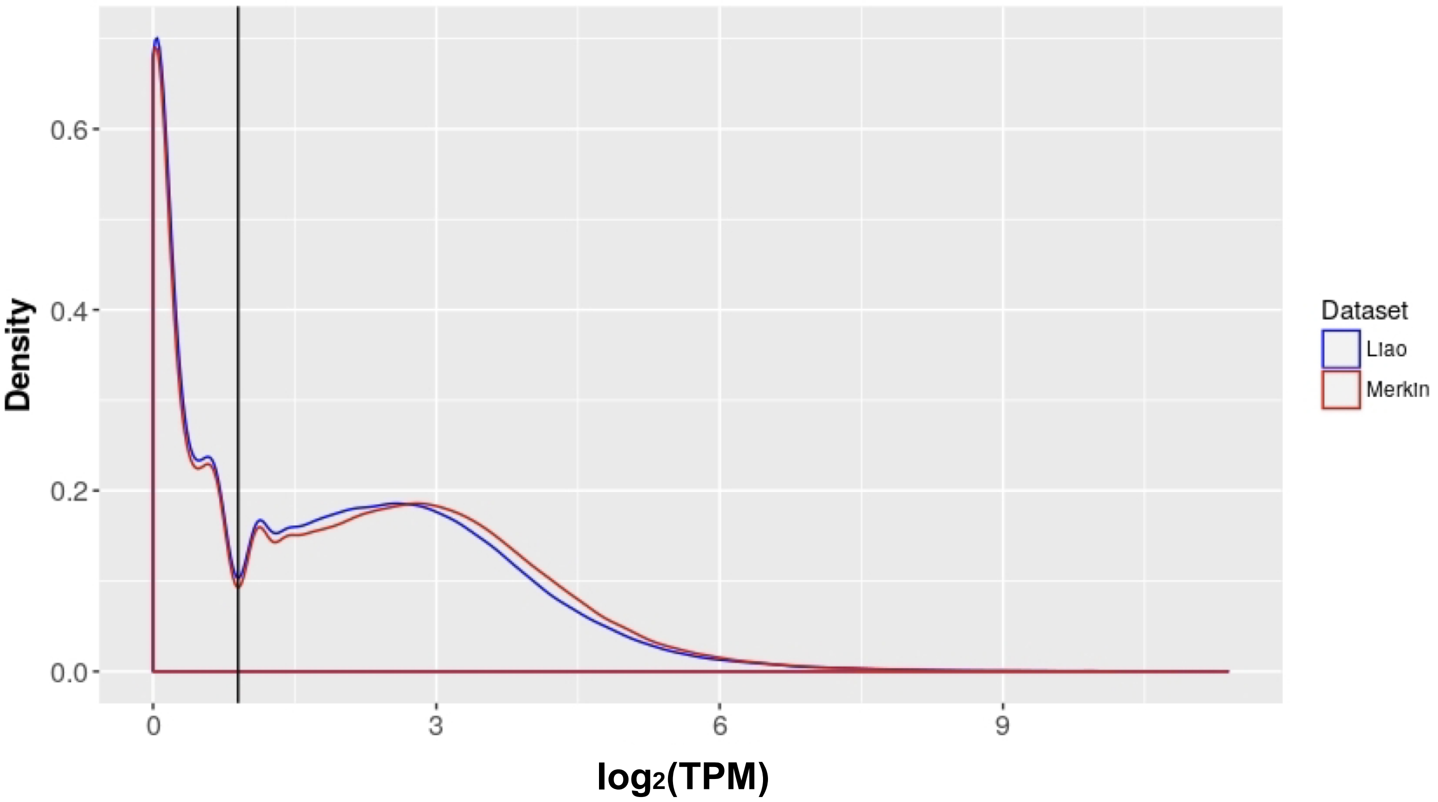

Supplement: S1 Fig — (PDF) [file pone.0216475.s001.pdf]
