# Supplementary material for: Computational identification of tissue-specific transcription factor cooperation in ten cattle tissues
Source: PLoS One. 2019 May 16;14(5):e0216475. doi: 10.1371/journal.pone.0216475 (PMC6522001; doi:10.1371/journal.pone.0216475)

Cooperation network for the TSG-set-specifc TF pairs of adipose tissue.

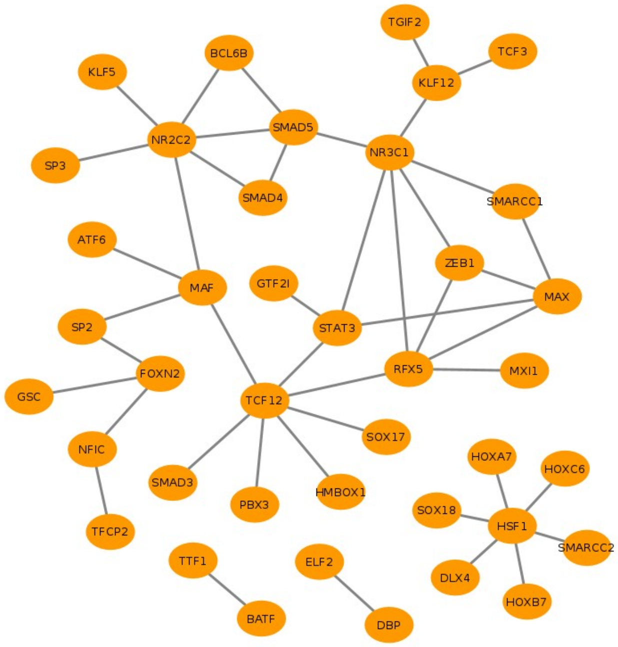

Cooperation network for the TSG-set-specifc TF pairs of colon.

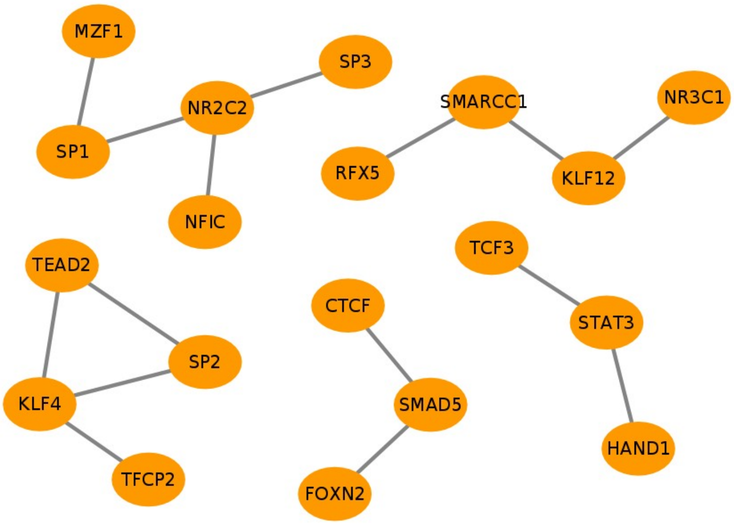

Cooperation network for the TSG-set-specifc TF pairs of duedenum.

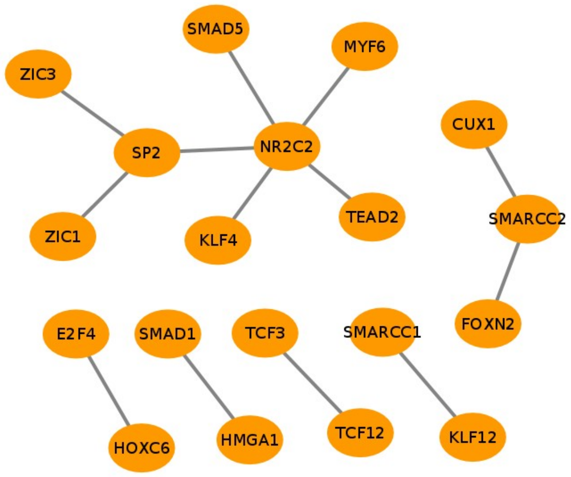

Cooperation network for the TSG-set-specifc TF pairs of heart.

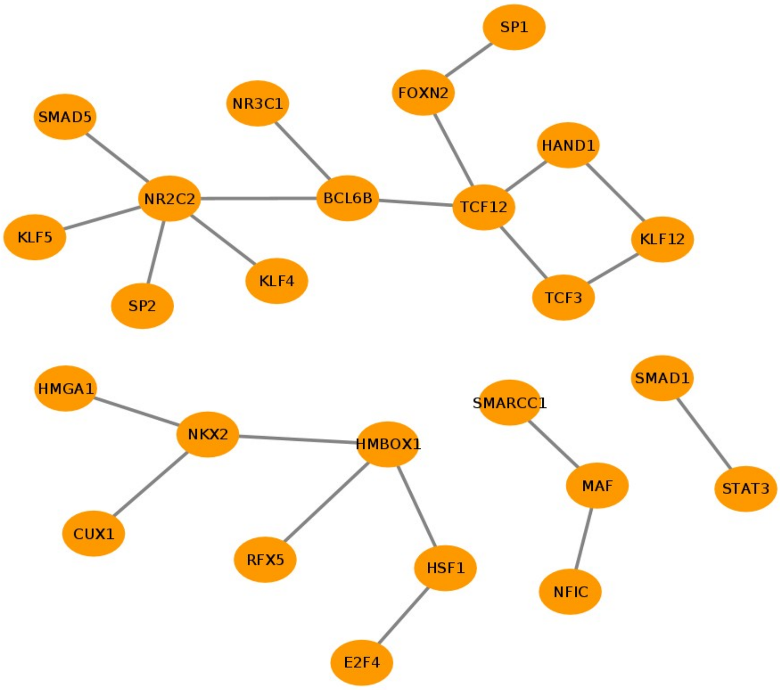

Cooperation network for the TSG-set-specifc TF pairs of muscle tissue.

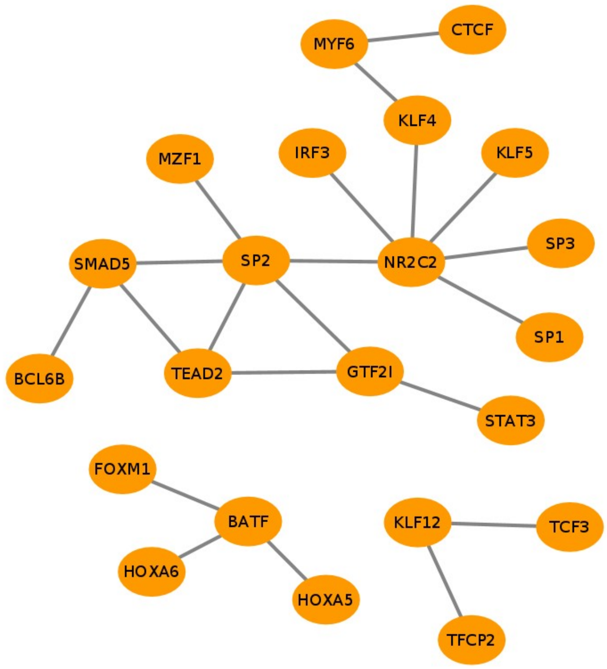

Cooperation network for the TSG-set-specifc TF pairs of spleen.

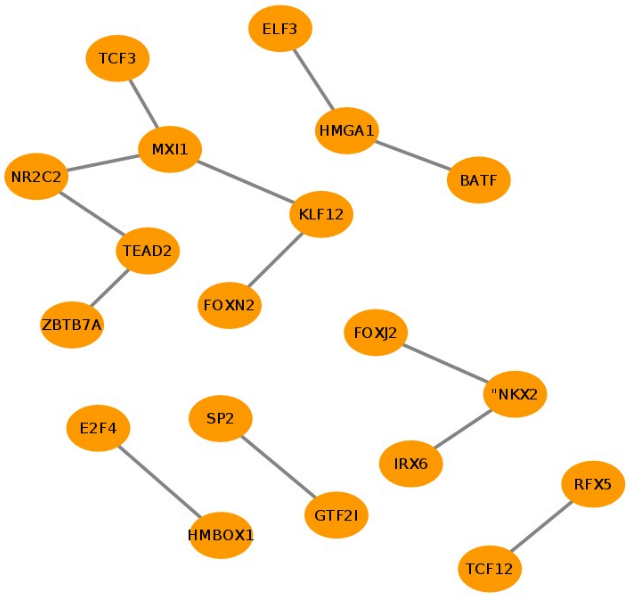

Cooperation network for the TSG-set-specifc TF pairs of testis.

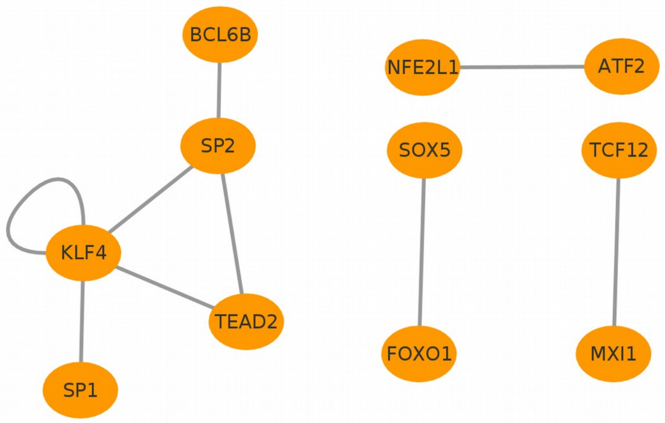

Supplement: S2 Fig — (PDF) [file pone.0216475.s002.pdf]
